# Discovery, Validation and Characterization of *Erbb4* and *Nrg1* Haplotypes Using Data from Three Genome-Wide Association Studies of Schizophrenia

**DOI:** 10.1371/journal.pone.0053042

**Published:** 2013-01-03

**Authors:** Zeynep Sena Agim, Melda Esendal, Laurent Briollais, Ozgun Uyan, Mehran Meschian, Luis Antonio Mendoza Martinez, Yongmei Ding, A. Nazli Basak, Hilmi Ozcelik

**Affiliations:** 1 Neurodegeneration Research Laboratory, Molecular Biology and Genetics Department, Bogazici University, Istanbul, Turkey; 2 Fred A. Litwin Centre for Cancer Genetics, Samuel Lunenfeld Research Institute, Mount Sinai Hospital, Toronto, Ontario, Canada; 3 Prosserman Centre for Health Research, Mount Sinai Hospital, Toronto, Ontario, Canada; The George Washington University, United States of America

## Abstract

Schizophrenia is one of the most common and complex neuropsychiatric disorders, which is contributed both by genetic and environmental exposures. Recently, it is shown that NRG1-mediated ErbB4 signalling regulates many important cellular and molecular processes such as cellular growth, differentiation and death, particularly in myelin-producing cells, glia and neurons. Recent association studies have revealed genomic regions of *NRG1* and *ERBB4*, which are significantly associated with risk of developing schizophrenia; however, inconsistencies exist in terms of validation of findings between distinct populations. In this study, we aim to validate the previously identified regions and to discover novel haplotypes of *NRG1* and *ERBB4* using logistic regression models and Haploview analyses in three independent datasets from GWAS conducted on European subjects, namely, CATIE, GAIN and nonGAIN. We identified a significant 6-kb block in *ERBB4* between chromosome locations 212,156,823 and 212,162,848 in CATIE and GAIN datasets (p = 0.0206 and 0.0095, respectively). In *NRG1*, a significant 25-kb block, between 32,291,552 and 32,317,192, was associated with risk of schizophrenia in all CATIE, GAIN, and nonGAIN datasets (p = 0.0005, 0.0589, and 0.0143, respectively). Fine mapping and FastSNP analysis of genetic variation located within significantly associated regions proved the presence of binding sites for several transcription factors such as SRY, SOX5, CEPB, and ETS1. In this study, we have discovered and validated haplotypes of *ERBB4* and *NRG1* in three independent European populations. These findings suggest that these haplotypes play an important role in the development of schizophrenia by affecting transcription factor binding affinity.

## Introduction

Schizophrenia [OMIM: 181500] is one of the most common neuropsychiatric disorders worldwide with a lifetime rate of 1% [1]. In several twin studies it has been proved that genetic heritability of schizophrenia is about 80%; however, environmental factors and de novo mutations also play key roles in developing the disorder [2]. Recently, the impact of ERBB4-NRG1 axis in schizophrenia has received great scientific attention. NRG1-mediated ErbB4 signalling regulates many important cellular processes such as growth, differentiation and death in various cell-types, particularly in myelin-producing cells, glia and neurons [3]. An earlier study has shown that mice heterozygous for *ERBB4* are associated with behavioural phenotypes of schizophrenia [4]. Several other studies have revealed that the disruption of NRG1-ErbB4 signalling leads to dysfunction in neuronal migration [5], NMDA hypofunction [6] and regulation of GABAergic neurotransmission [7] that are also disrupted in schizophrenia. Additionally, phosphorylation of Erbb4 by NRG1 and downstream AKT and ERK2 signalling are more likely to be activated in schizophrenia, compared to control samples [6].

Several studies have investigated the impact of single nucleotide polymorphisms (SNPs) and haplotypes of *ERBB4* and *NRG1* on the risk of developing schizophrenia. With respect to *ERBB4* haplotypes, a 3-SNP haplotype (rs707284, rs839523, rs7598440) surrounding exon 3 has been identified in individuals with Ashkenazi Jewish background [8]. These findings have been validated in three independent populations as reported in a study by Nicodemus and colleagues, where they have also identified another 3-SNP haplotype (rs3748962, rs2289086 and rs3791709), flanking intron 23 - exon 27 at the 3′ end of *ERBB4* [9]. A larger Scottish study has shown that 14 out of 109 SNPs in the *ERBB4*, mostly located at the two ends of the gene, are significantly associated with schizophrenia according to both allelic and genotypic genetic models, but a consistent pattern could not be observed [10]. The major risk haplotype of *NRG1* at chr8: 31,475,521–31,785,232 (also termed as Hap_ICE_), represented a five SNP haplotype (SNP8NRG221132, SNP8NRG221533, SNP8NRG241930, SNP8NRG243177 and SNP8NRG422E1006) at the 5′ end of *NRG1* is identified in the Iceland population [4]. The *NRG1* haplotypes encompassing the Hap_ICE_ and nearby regions in various populations have been extensively reviewed by Walker et al. [11], and in several meta-analysis studies [12], [13], [14]. An alternative region at the 3′ end of the gene at chr8: 32,600,000–32,800,000, has been also identified and validated in bipolar disorder as well as in schizophrenia [15].

In this study, we aim to validate and define potential risk-associated haplotypes of *ERBB4* and *NRG1* in schizophrenia through a systematic analysis of SNP genotype data from three published genome-wide association studies (GWAS) , CATIE, GAIN and nonGAIN, all of which have large (>500) case-control samples with Caucasian origin.

## Methods

### Study populations and design

The genotype data for cases and controls was obtained from Database of Genotypes and Phenotypes (dbGaP) (http://www.ncbi.nlm.nih.gov/sites/entrez?db=gap) [16]. The samples that included subjects with Caucasian origin were collected from (a) GAIN (Genetic Association Information Network) dataset [dbGAP accession number: phs000021.v2.p1] consisted of 1110 cases and 1107 controls and genotyped for 906,600 SNPs [17]; (b) non-GAIN dataset [dbGAP accession number: phs000167.v1.p1] with 989 cases and 1096 controls genotyped for 909,622 SNPs [17]; and (c) CATIE (Clinical Antipsychotic Trials of Intervention Effectiveness) including 414 cases and 414 controls genotyped for 495,172 SNPs (after removing African American subjects and eliminating individuals who are older than 65 years of age in order to match population characteristics of GAIN and non-GAIN datasets) [18].

Both the CATIE study and the Molecular Genetics of Schizophrenia (MGS) study, including the GAIN and nonGAIN datasets have used the Diagnostic and Statistical Manual of Mental Disorders criteria for the diagnosis of schizophrenia patients. While in the CATIE study, only patients with schizophrenia were included, the MGS study group consisted of additional schizoaffective disorder patients (∼%10) who had symptoms similar to schizophrenia for at least six months.

### Systematic analysis to identify novel haplotypes

The GWADview software is a visualization tool, designed by Ozcelik Lab, to interpret GWAS results. The analysis is based on various algorithms such as allelic, genotypic, dominant and recessive test models. The system provides a single, integrated plot of SNP distribution according to physical location on the chromosome and p-values from multiple resources. Using this platform, we investigated the distribution of associated SNPs and more importantly SNP clusters along the gene sequence. The genotype data from regions of lowest p-values observed for at least two datasets was retrieved for haplotype-based analyses using PLINK software (http://pngu.mgh.harvard.edu/purcell/plink/) [19]. Since CATIE and GAIN/nonGAIN datasets used different arrays with different numbers and sets of SNPs, we focused our analysis on common SNPs, 122 in *ERBB4* and 193 in *NRG1*, found in all three datasets. PLINK software was used to perform haplotype-based logistic regression model for calculating p-value and odd's ratio (OR) of each haplotype. To compare haplotype blocks, we used the block structure of each dataset to investigate all datasets, thus avoiding the misinterpretation of findings due to ethnic interbreeding. Since results of the three block structure testing were very similar, we used the CATIE block structure from all three datasets for our SNP analysis for convenience. The SNPs included were refined to those which agreed with Hardy-Weinberg equilibrium of >0.05 and minor allele frequency of >0.1. Haploview software was also used to visualize haplotype blocks within significant regions found by GWAS (http://www.broad.mit.edu/haploview/haploview) [20]. We have utilized the MaCH software [21] to carry out the imputation analysis. RSQ score estimates the correlation between imputed and true genotypes. Since RSQ scores were very low when 1000genome data were imputed, instead we used HapMap data (release 27) to impute genotype data of common (MAF>10%) and rare SNPs (MAF<10%) in the *ERBB4* and *NRG1* blocks associated with schizophrenia via haplotype analysis.

### Validation of previously identified haplotypes

Several *ERBB4* and *NRG1* polymorphisms and haplotypes are previously reported to be significantly associated with the risk of developing schizophrenia in various ethnic populations. Since only a few of the previously identified variants matched with SNPs available in our datasets, the majority of the validation was done using other SNPs in linkage disequilibrium (LD) which were identified by the online SNP Annotation and Proxy Search (SNAP) tool [22]. The threshold for R^2^ has been set to 0.8 with a distance maximally confined to 500 kb in HapMap CEU (Utah residents with Northern and Western Europe ancestry) sample. The proxy SNPs in strong LD with disease-associated SNPs was analysed using Haploview. For *NRG1*, only the regions that have been validated more than twice became further validated in three GWAS datasets.

### Fine mapping

The regions that were found to be significant in more than one dataset in our study were also subject to fine mapping for the purpose of identifying transcription factor binding sites (TFBS). All SNPs within candidate regions of *ERBB4* and *NRG1* were present in the Build 132 version of dbSNP database (http://www.ncbi.nlm.nih.gov/projects/SNP/). Transcription factors (TFs) that bind to major and minor allele for each polymorphism were identified using FastSNP software (http://fastsnp.ibms.sinica.edu.tw) [23]. Subsequently, we pursued our analysis of possible TF interactions with regions containing significant haplotypes in CATIE, GAIN and nonGAIN datasets.

## Results

In this study, we utilized a haplotype-based approach to systematically screen for novel associations, as well as to validate the previously reported variations within *ERBB4* and *NRG1* that conferred risk of developing schizophrenia. Our analyses were based on SNPs from three European Caucasian populations of schizophrenia GWAS datasets, CATIE, GAIN and non-GAIN (Table 1).

**Table 1:** Summary of *ERBB4* and *NRG1* variants in GWAS datasets.

				Number of SNPs			
Gene Symbol	Chromosome Number	Start position	End position	CATIE	GAIN	nonGAIN	Common
*ERBB4*	2	211,948,687	213,111,597	226	368	346	122
*NRG1*	8	31,616,810	32,720,310	262	439	472	193

Chromosome location, according to NCBI Build 36, of *ERBB4* and *NRG*1 are shown in the table. SNPs were pooled after Hardy-Weinberg and minor allele frequency cut-off that are 0.05 and 0.001, respectively.

### Novel haplotypes of *ERBB4* and *NRG1* loci

**Figure 1:**
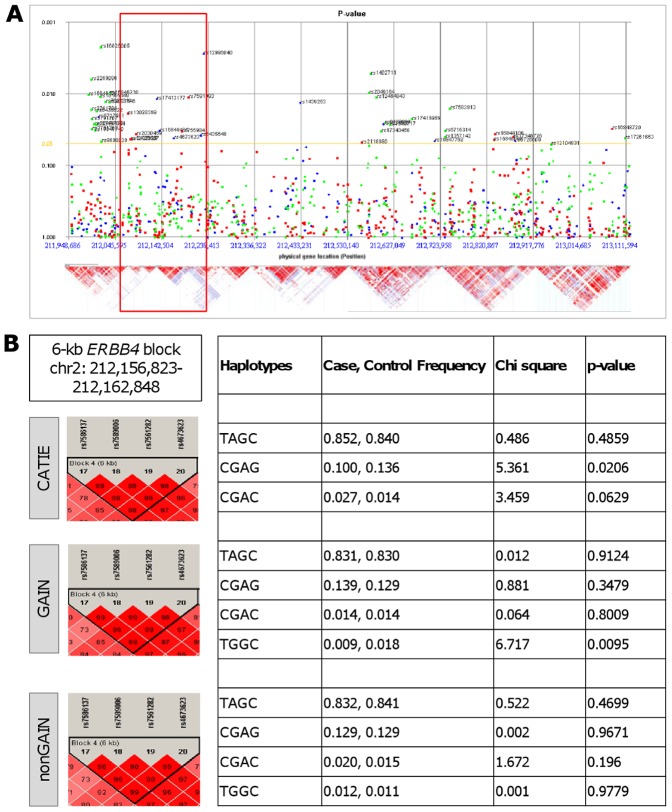
Validation of previously identified and identification of novel haplotypes of *ERBB4* in schizophrenia GWAS datasets. **A.** *ERBB4* polymorphims in three schizophrenia GWAS datasets are illustrated in GWADview software. SNPs are plotted by their location on the y-axis and by their genomic position on the x-axis. Blue represents CATIE, red GAIN and green nonGAIN. The lower panel shows haplotype blocks of this region in Hapmap CEU population. **B.** LD plots of *ERBB4* with the most significant haplotypes in the region from 212,100,000 bp to 212,200,000 bp. Cut-off value for Hardy-Weinberg is 0.05 and for minor allele frequencies 0.001.

Using the GWADview tool, we initially identified a 200-kb region within the genomic region of *ERBB4* at chr2: 212,050,000–212,250,000, which included a total of 11 significant (p<0.05) SNPs from CATIE and GAIN datasets (Figure 1A). The significance of this region also confirmed using haplotype-based logistic regression test and it was observed that other regions did not reveal any significant results (data not shown). Further refinement of the region using logistic regression and the Haploview analysis has identified a 6-kb haplotype block at chr2:212,156,823–212,162,848, to be significantly associated with schizophrenia risk in CATIE and GAIN datasets (Figure 1B). The most significant haplotype corresponding to this 4-SNP block (rs7586137-rs7589006-rs7561282 and rs4673623) included the C-G-A-G haplotype (MAF_case_ = 0.100, MAF_control_ = 0.136, p = 0.0206) in the CATIE study. The same block highlighting a different haplotype (T-G-G-C) was found to be significant in the GAIN dataset (MAF_case_ = 0.009, MAF_control_ = 0.018, p = 0.0095). The C-G-A-C haplotype was not significantly associated in the nonGAIN dataset (p = 0.196).

**Figure 2:**
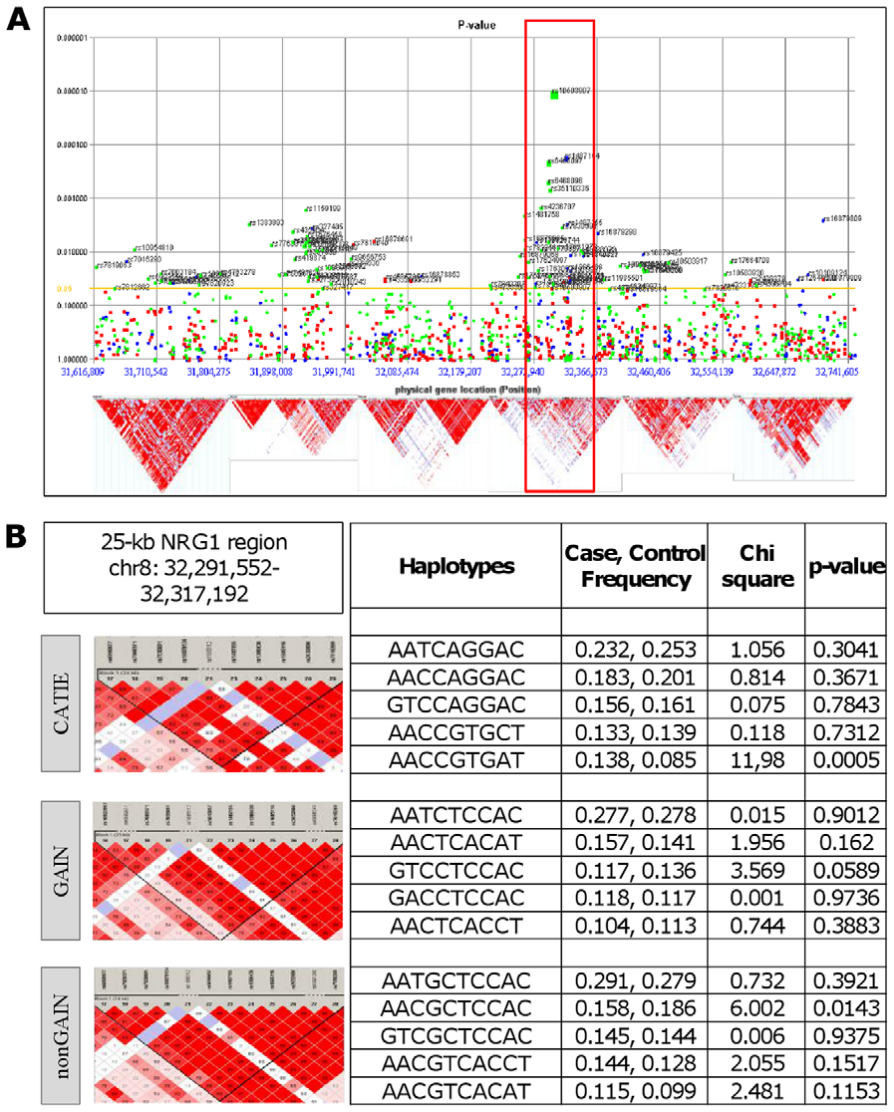
Validation of previously identified and identification of novel haplotypes of *NRG1* in schizophrenia GWAS datasets. **A.** *NRG1* polymorphims in three schizophrenia GWAS datasets are illustrated in GWADview software. SNPs are plotted by their location and genomic position on the y and x-axis respectively. Blue represents CATIE, red GAIN and green nonGAIN. The lower panel shows haplotype blocks of this region in Hapmap CEU population. **B.** LD plots of *NRG1* with the most significant haplotypes in the region from 32,250,000 bp to 32,400,000 bp. Cut-off value for Hardy-Weinberg is 0.05 and for minor allele frequencies 0.001.

With respect to *NRG1*, GWADview analysis has revealed a genomic region on chr8 within a 150 kb region (32,250,000–32,400,000) where SNPs, from three datasets, with low p-values were also identified (Figure 2A). Subsequent Haploview and logistic regression analysis identified a 25-kb region at chr8: 32,291,552–32,317,192 in CATIE, GAIN and nonGAIN that also consisted of the most significant SNPs (rs10503907 and rs1487155), observed in GWADview. The most significant haplotype of this block, found in CATIE, is the A-A-C-C-G-T-G-A-T haplotype, which was overrepresented in cases over controls (MAF_case_ = 0.138, MAF_control_ = 0.085, p = 0.0005). The G-C-T-C-C-T-C-C-A-C haplotype was marginally significant in GAIN (MAF_case_ = 0.117, MAF_control_ = 0.136, p = 0.0589), while the A-A-C-G-C-T-C-C-A-C haplotype, residing in approximately 16% of cases and 19% of controls, was significant in the nonGAIN dataset (p = 0.0143) (Figure 2B).

### Imputation analysis

To improve genotyping rate (i.e. missing genotypes) and also to identify additional SNPs within the associated haplotype blocks of *ERBB4* and *NRG1*, we have carried out imputation analysis using the MaCH software and HapMap data. Table S1 summarized minor allele frequencies and p-values of SNPs before and after imputation.

As a result, one additional SNP (rs1818571) was found within the *ERBB4* block after imputation. We performed haplotype association using five SNPs and compared the results with the pre-imputation haplotype analysis. While C-G-A-T-C haplotype had the same p-value (p = 0.0206) as the C-G-A-C haplotype in the pre-imputed CATIE dataset, in GAIN and nonGAIN, the significance of the block T-G-G-C-C improved with p-values of 10^−9^ and 5×10^−6^, respectively (Table S2).

Imputation analysis of 25-kb haplotype block of *NRG1* revealed 46 SNPs (35 additional SNPs) in the same region. In CATIE, four blocks of the candidate region had significant haplotypes with p-values varying from 0.05 to 0.0001 (Table S3). In the GAIN dataset, a single 1-kb haplotype within the candidate region, C-G-C-T-C-G-A-G-T, revealed stronger association with schizophrenia (p = 0.00457) compared to the pre-imputation haplotype. However, imputation analysis did not improve the significance of the candidate *NRG1* haplotypes (p_6-kb haplotype_ = 0.0424 and p_12-kb haplotype_ = 0.0339) in the nonGAIN dataset.

### Gender-specific haplotypes of *ERBB4* and *NRG1*

After we defined the significant haplotype blocks in *ERBB4* and *NRG1* in all three datasets, we have investigated the gender-specific associations. Although we could not observe any significant association for *ERBB4* in female and male datasets, *NRG1* has shown gender-specific significant correlations.

**Figure 3:**
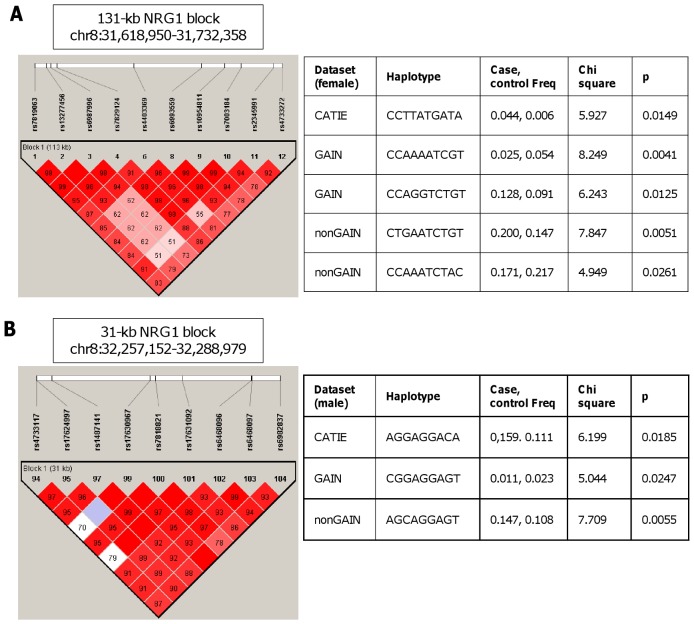
Identification of gender-specific association of *NRG1* in schizophrenia GWAS datasets. LD plots of *NRG1* with the most significant haplotypes **A.** in the region from 31,618,950 bp to 31,732,358 bp in females. **B.** in the region from 32,257,152 bp to 32,288,979 bp in males Cut-off value for Hardy-Weinberg is 0.05 and for minor allele frequencies 0.001.

In female datasets, a 131-kb haplotype block located between 31,618,950 bp and 31,732,358 bp was found to be significantly associated with schizophrenia (Figure 3A). The significant haplotype corresponding to this 10-SNP block included C-C-T-T-A-T-G-A-T-A in the CATIE dataset (MAF_case_ = 0.044, MAF_control_ = 0.006, p = 0.0149). While the most significant haplotype in the GAIN dataset was C-C-A-A-A-A-T-C-G-T (MAF_case_ = 0.025, MAF_control_ = 0.054, p = 0.0041), a different haplotype of the same block was in strong association with schizophrenia in female nonGAIN study group (C-T-G-A-A-T-C-T-G-T, MAF_case_ = 0.200, MAF_control_ = 0.147, p = 0.0051).

A 31-kb *NRG1* haplotype block between 32,257,152 bp and 32,288,979 bp, representing A-G-G-A-G-G-A-C-A haplotype, was found to be significant in the male CATIE dataset with a p-value of 0.0185 (MAF_case_ = 0.159, MAF_control_ = 0.111). The C-G-G-A-G-G-A-G-T haplotype that was carried by 1% of male cases and 2% of male controls was significant in GAIN (p = 0.0247), while the A-G-C-A-G-G-A-G-T haplotype (MAF_case_ = 0.147, MAF_control_ = 0.108, p = 0.0055) confirmed the significance of this block in nonGAIN (Figure 3B).

### Previously identified haplotypes of *ERBB4* and *NRG1* loci

The first 3-SNP haplotype (rs707284-rs839523-rs7598440) surrounding exon 3 of *ERBB4* was found to be significantly associated with schizophrenia in Ashkenazi Jewish population [8]. The same haplotype failed to reach significance in our study, yet, implicated a borderline effect in GAIN (p = 0.0793) and nonGAIN (p = 0.0527) datasets. The other 3-SNP haplotype (rs3748962-rs2289086-rs3791709) flanking the region in intron 23 to exon 27 [9], was also validated in our study using solid spine of LD method (D′>0.6) in the Haploview software (Table 2). The results were based on the significant p-values of 0.03, 0.048 and 0.034 obtained in CATIE, GAIN and nonGAIN study datasets respectively.

**Table 2:** Previously identified regions and their validations in our study.

	Location^a^	Flanking regions	Associated p-value^b^	References	Proxy etc. information^c^	p-value CATIE^d^	p-value GAIN^d^	p-value nonGAIN^d^
***ERBB4***								
3-SNP (rs707284/rs839541-rs839523-rs7598440) haplotype	212,501,443–212,547,291/212,524,334	exon 3	0.00044/0.032	[8], [9]	R^2^>0.8, H3, 10 kb, CEU	No SNPs	0.0793	0.0527
3-SNP (rs3748962-rs2289086-rs3791709) haplotype	211,960,109–211,993,348	intron 23- exon 27	0.020	[9]	R^2^>0.8, T, H3, 10 kb, CEU	0.03	0.048*	0.034
***NRG1***								
5′ Region, The Hap_ICE_ haplotype	31,475,521–31,785,232	exon 1 and 5′ of intron 1	0.0001–0.05	[11]	Haploview	0.0166**	0.0011**	0.0448**
3′ Region	32,600,000–32,800,000	several exons	0,0001–0.005	[11]	Haploview	0.0008**	0.0003**	0.0078**

a : NCBI Build 36.

b : p-value of the most significant haplotype from paper.

c : SNAP search criteria: R2>0.8 SNP dataset: T (1000 Genome Pilot 1), H3 (Hapmap3), Distance limit: 10 kb, Population: CEU.

d : p-value of the most significant haplotype block with SNPs associated with previously identified haplotypes. P-values are calculated by Haploview with the Solid Spine analysis method (D′>0.6).

* : p-values are calculated by creating custom blocks using Haploview software.

** : p-values are calculated using the Haploview solid spine method (D′>0.6). The most significant haplotype block of region B is different in CATIE, GAIN and nonGAIN.

Both of the *NRG1* haplotypes previously located at 5′ (chr8: 31,550,000–31,850,000) and 3′ regions (chr8: 32,600,000–32,800,000) were also validated in all three GWAS datasets used in our study. A relatively strong association of the 3′ region haplotype (p_CATIE_ = 0.0008, p_GAIN_ = 0.0003 and p_nonGAIN_ = 0.0078) was identified when it was compared to the 5′ Hap_ICE_ haplotype (p_CATIE_ = 0.0166, p_GAIN_ = 0.0011 and p_nonGAIN_ = 0.0448) (Table 2).

### Fine mapping using Genome Browser and FastSNP analysis

For further analyses of the novel *ERRB4* and *NRG1* haplotypes, TFs, which bind to major and minor alleles for each SNP, were identified using FastSNP software. All SNPs between these regions were listed in the dbSNP database (Build 132 version). The summary of this analysis is shown in Table 3.

**Table 3:** FastSNP analysis summary of SNPs in defined region of *ERBB4* and *NRG1*.

					Identified SNPs		
Gene	First SNP	Last SNP	Number of SNPs	Unidentified SNPs	Common SNPs	Rare SNPs	Very Rare SNPs
*ERBB4*	rs7586137	rs4673623	63	29	13	9	12
*NRG1*	rs10503907	rs7016269	317	122	74	27	94

The frequencies for common, rare and very rare SNPs are >25%, 10%-1% and <1%, respectively.

**Figure 4:**
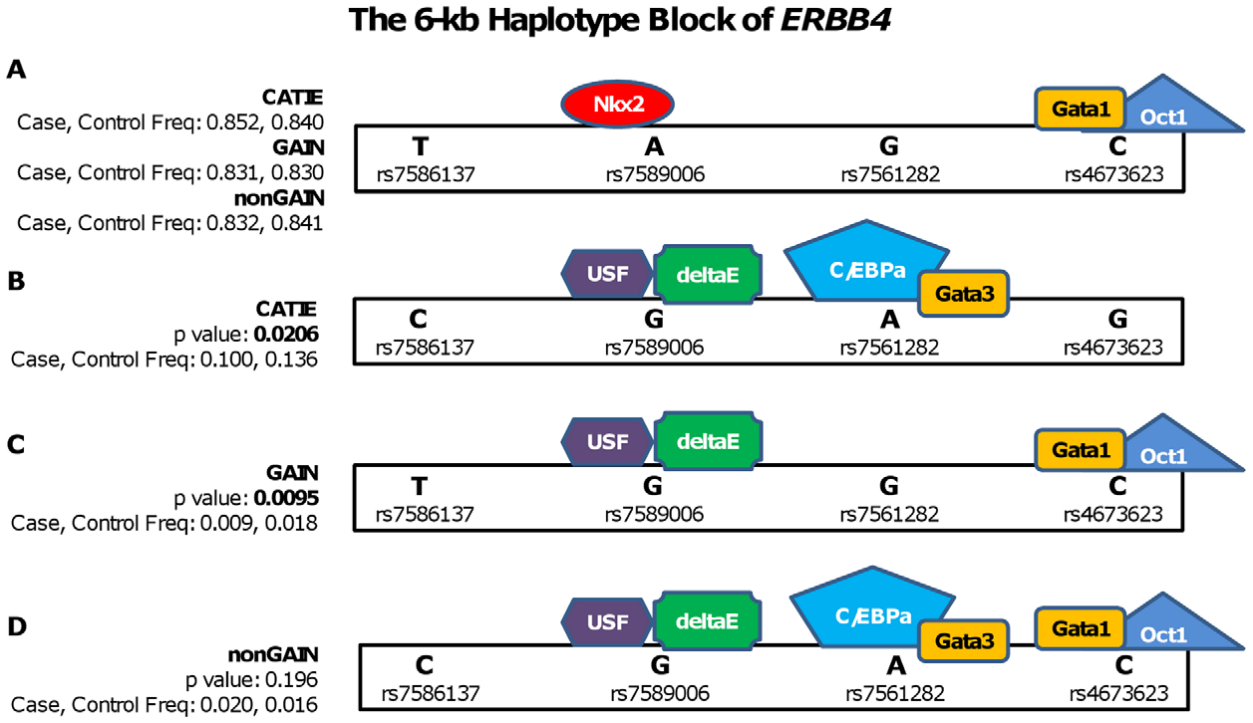
Transcription factors that bind common and significant haplotypes of *ERBB4*. The frequencies and p-values of transcription factors that bind to the 6-kb haplotype block of *ERBB4* (chr 2: 212,156,823–212,162,848) in CATIE, GAIN and nonGAIN datasets are illustrated. **A.** The most common haplotype of 6-kb block in CATIE, GAIN and nonGAIN, T-A-G-C, and transcription factors that bind to this haplotype are shown. **B**, **C** and **D** depict the significant haplotypes of the same *ERBB4* block in CATIE, GAIN and nonGAIN respectively.

Using dbSNP database, a total of 63 SNPs, including the four tagged-SNPs (rs7586137-rs7589006-rs7561282-rs4673623) detected here, were identified within the novel 6 kb *ERBB4* haplotype block at chr2:212,156,823–212,162,848. FastSNP analysis of all the identified SNPs showed that the most common T-A-G-C haplotype allele (MAF≈0.80) found in three GWAS datasets, facilitated the simultaneous binding of NKX2 and GATA1/OCT1 transcription complex at the rs7589006 (allele A) and rs4673623 (C allele) loci, respectively (Figure 4A). The significant haplotypes of CATIE (MAF_case_ = 0.100, MAF_control_ = 0.136, p = 0.0206) and GAIN (MAF_case_ = 0.009, MAF_control_ = 0.018, p = 0.0095) were found to be overrepresented in controls, suggesting a protective effect against schizophrenia. The major difference between the common haplotypes and the relatively less common, yet, significant haplotypes was the replacement of the TF site for NKX2 with USF and deltaE at rs7589006 (G instead of A allele) (Figure 4B–C). The SNPs, rs7561282 and rs467362, which are the last 2 markers of a 4-SNP haplotype, bind to C/EBPa/GATA3 and GATA1/OCT1, respectively. Both sites seemed to be occupied in the nonGAIN haplotype allele C-G-A-C (p = 0.196) (Figure 4D). Since none of the haplotypes were able to achieve the significance level (p<0.05) for the nonGAIN dataset, we could not conclude any transcription factor binding.

**Figure 5:**
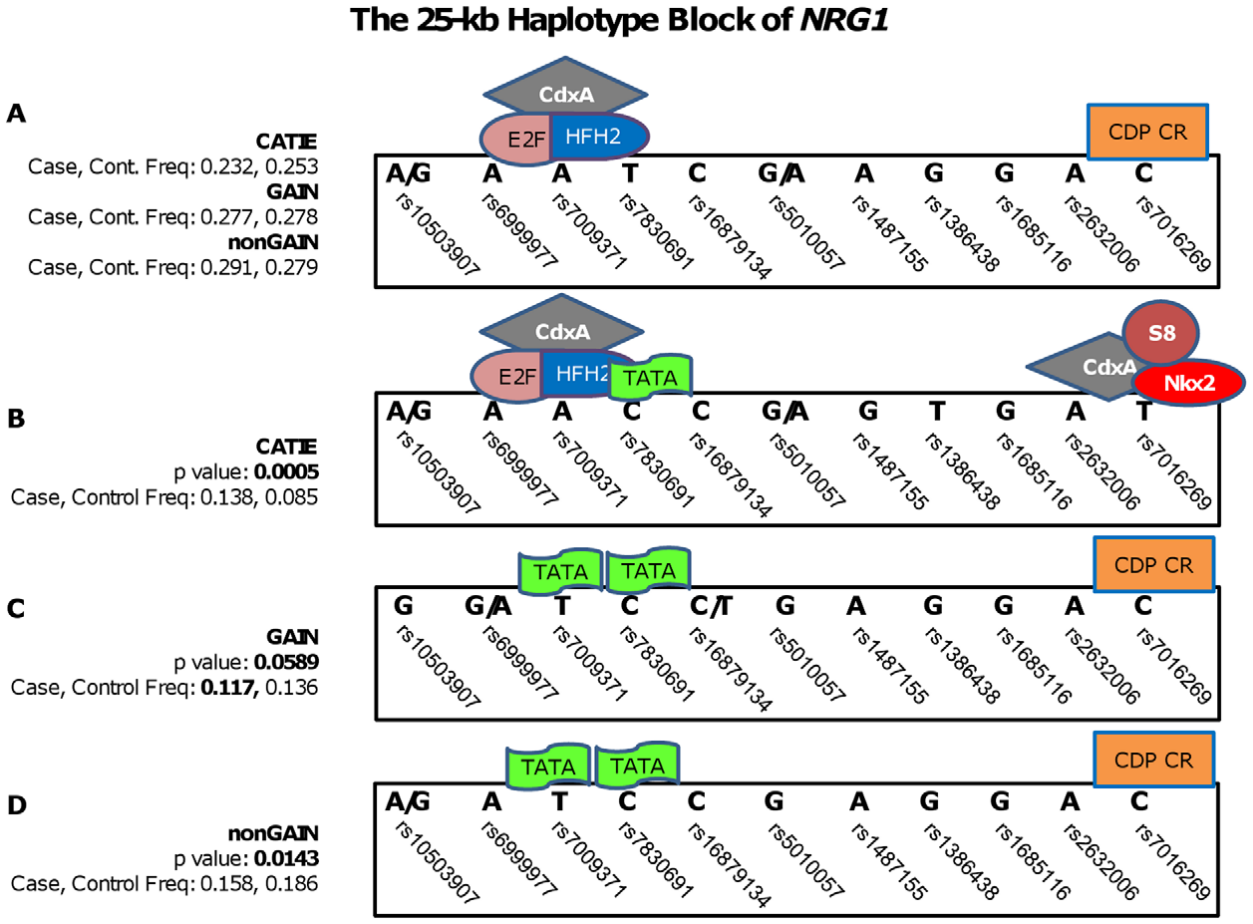
Transcription factors that bind common and significant haplotypes of *NRG1*. The frequencies and p-values of transcription factors that bind to the 25-kb haplotype block of *ERBB4 NRG1* block (chr 8: 32,291,552–32,317,192) in CATIE, GAIN and nonGAIN datasets are illustrated. **A.** The most common haplotype of 25-kb block in CATIE, GAIN and nonGAIN, and transcription factors that bind to this haplotype are shown. **B**, **C** and **D** depict the significant haplotypes of the same *NRG1* block in CATIE, GAIN and nonGAIN respectively.

Within the significant 25 kb *NRG1* block at chr8:32,291,552–32,317,192, we retrieved a total of 317 SNPs, 11 of which were found at least in one of the haplotypes of CATIE, GAIN and nonGAIN datasets. FastSNP analysis revealed that the combination of SNPs represented within the haplotypes, resulted in the alteration of TFBS, including E2F, CDXA, HFH2, TATA, NKX2, CDP CR and S8. The common alleles of the 11-SNP haplotype in all datasets facilitated the binding of CDXA, E2F, HFH2 complex when rs7009371 expressed A allele and simultaneously the binding of the CDP CR at rs7016269, when it expressed the C allele (Figure 5A). The A/G-A-A-C-C-G/A-G-T-G-A-T haplotype in CATIE, which conferred a significantly increased risk, facilitated the binding sites suitable for CDXA, E2F, HFH2 (rs7009371, A allele) and S8, NKX2 and CDXA (rs7016269, T allele) (Figure 5B) Unlike CATIE, the significant haplotypes in GAIN and nonGAIN were found to have protective effects. The significant haplotypes in GAIN and nonGAIN showed increased binding affinity for TATA (rs7009371, T allele) and for CDP CR (rs7016269, C allele). The rs7009371 and rs7016269 resulted in binding of TATA and CDP CR, except the ones in CATIE. (Figure 5C–D)

### Rare SNPs and haplotypes

FastSNP analyses of rare SNPs that were annotated in dbSNP, yet, not validated, suggested alterations of more critical TFBS. Among the *NRG1* variants some (A) abolished an existing TFBS such as rs13262178 [C binds NRF-2; T binds none], rs34393015 [wt binds Sox5-HFH2; 6pb insertion binds none], rs58045757 [G binds HNF-3b; delG binds none], rs34158863 [wt binds c-ETS; insG binds none] and rs34782215 [wt binds c-ETS, ELK1; insG binds none]; (B) Create a new site such as rs12680997 [C binds none; T binds SRY], rs13278702 [G binds none; T binds GR] and rs34985716 [G binds none; T binds Sox5-SRY] and; (C) Replaced an existing site with binding sites of new TFs such as rs66776820>6 bp [TATA/E2F-CdxA, HFH2-Evi1], rs71832406>6 bp [S8/CdxA], rs35422231 [A binds CEBP-CDxA, C binds ARP1] and rs71512619 A/C [A binds deltaE-AML1a, and C binds MZF1]. Among the few rare SNPs identified within ERBB4 region included rs13012759 [A binds CdxA; G binds none], rs12989265 C/T [C binds none; T binds SRY-Sox5], rs12989282 [C binds none; T binds SRY], and rs58786592 A/C [A binds c-ets; C binds Gata1-Gata2]. These SNPs are listed in Table S4.

## Discussion

Many studies investigated the genetic association of *NRG1* and *ERBB4* with a risk of developing schizophrenia in various ethnic groups [8], [11]. In this study, we systematically analyzed novel and previously identified genetic associations of *NRG1* and *ERBB4* using filtered European datasets from three GWAS. Having obtained promising results, our approach has been proven to be effective due to (A) multiple datasets from age and sex-matched, large size of schizophrenia and healthy control samples filtered for homogenous European ethnicities; (B) dense and quality SNP markers of *ERBB4/NRG1* with an average 99% for genotyping call rate for SNPs and individuals. (C) a comprehensive genetic analyses where we systematically applied various haplotype-based association methods including GWADView, logistic regression model, and Haploview analyses for the refined region.

### Validation of the previously identified regions

The first 3-SNP haplotype of *ERBB4* (rs707284-rs839523-rs7598440), surrounding exon 3, was found to be previously associated with schizophrenia in the Ashkenazi population [8]. However, they were borderline-significant (0.05<p<0.1) in GAIN and nonGAIN datasets. The 3-SNP G-A-A haplotype (rs3748962-rs2289086-rs3791709) flanking intron 23 - exon 27 [9], was also validated in the three datasets in our study: 296 families from Clinical Brain Disorders Branch/National Institute of Mental Health Sibling Study were compared with 370 healthy controls in family-based affection analyses. While this study identified a G-A-A haplotype at p-value of 0.02 significance, we found the similar region to be significant (0.03<p<0.048) in all three datasets. Since the same SNPs were not assayed in these GWAS datasets, we cannot construct the exact haplotypes in these datasets. The same haplotype has been shown to be significant in the Han Chinese case-control study (CTA haplotype, p = 0.02, case vs. control = 36% vs 24%) [24]. rs3748962, causing a synonymous variant in exon 27 (Val1065Val), was implicated to play a role in variable mRNA expression of maternal and paternal chromosomes in the brain [25]. Although the haplotype was re-validated in three independent European populations in our study, *in vivo* or *in vitro* studies are necessary to reveal its effect and possible cis-acting element in ErbB4-NRG1 signalling in the mechanism underlying schizophrenia.

The studies that have showed an association between *NRG1* and schizophrenia risk mainly focused on two genomic regions, region A (Hap_ICE_) [11] and region B (32,600,000 bp–32,800,000 bp). Hap_ICE_ haplotype, located at the 5′ end of the gene covering exon 1 and the 5′ of intron 1, were also validated in our study [4]. The second region, Region B, covered most of the exons of *NRG1* which were concentrated at the 3′ end of the gene as reported in Walker et al [11]. Several studies have identified different haplotype blocks and polymorphisms in region B as significant in various schizophrenia populations [15], [26], [27]. Haploview analysis in our study implicated that different blocks in region B of *NRG1* were significant in three Caucasian GWA datasets. Although the 5′ end of the *NRG1* gene, including the Hap_ICE_ haplotype and other haplotypes nearby, has been shown to be significant in different ethnic schizophrenia SCZ populations, our study supported the role of the 3′ end of the *NRG1* gene on the prevalence of schizophrenia in European populations, a finding which has been suggested in only a few studies to date. [11]

### Identifying novel regions

Here, we have identified a novel 6-kb haplotype block in *ERBB4* on chr2 between the positions, 212,156,823 and 212,162,848, which was significantly associated with risk of developing schizophrenia in the Caucasian samples of CATIE and GAIN datasets. This 4-SNP haplotype block, consisting of rs7586137, rs7589006, rs7561282 and rs4673623, is significantly overrepresented in controls compared to schizophrenia patients. Although the significant haplotypes of 4-SNP block of *ERBB4* were different in CATIE and GAIN datasets, they both conferred protective role. We have also imputed the genotype data for both *ERBB4*. Interestingly, imputation resulting in five SNPs, improved the significance of haplotypes in GAIN and nonGAIN , while the CATIE dataset remained the same. This novel 6-kb block, located within intron 19 of *ERBB4*, is part of a genomic region encoding tyrosine kinase domain (known as the catalytic domain) (Uniprot ID: Q15303) that spans a region from exon 18 to exon 24. Although this haplotype did not affect coding region directly, it may lead to altered efficiency of the splicing mechanism via altering sites for TFs and thus, resulting in modified transcription efficiency. On the other hand, the analysis of female and male datasets did not result in any gender-specific association of *ERBB4* haplotype blocks.

The novel 25-kb haplotype block (11 SNPs) of *NRG1* was found to be located on chr8 between the positions 32,291,552 to 32,317,192. The significant haplotype in the CATIE dataset was overrepresented in cases, while significant haplotypes of the same block in GAIN and nonGAIN were more prevalent in controls than in cases. Haplotype analysis of 46 SNPs imputed has shown stronger association in GAIN, whereas CATIE and nonGAIN did not change, when compared with the results of pre-imputation analysis. This block, which is located within the intron 1 of *NRG1*, is likely to impact on promoter-enhancer sequences that are part of first introns of genes. Our analysis highlights the importance of this particular *NRG1* block in schizophrenia, such that the combination of different alleles of these SNPs within the corresponding block might have an impact on the regulation of *NRG1* gene expression. Moreover analysis of female and male datasets has revealed a significant association of a 131-kb haplotype block between 31,618,950 bp and 31,732,358 bp only in females. This novel block spans the 5′ untranslated region of *NRG1*, exon 1 and the beginning of intron 1 and may impact the regulation of *NRG1* gene expression. On the other hand, another block of *NRG1* between 32,257,152 bp and 32,288,979 bp was found to be in strong association in male subjects in all three datasets. This haplotype block corresponds to a 31-kb region that is close to the 3′ end of the first intron, which is predicted to be responsible to alter the accessibility of chromatin to transcription, thus potentially impacting gene expression [28].

### Mapping transcription binding sites altered within the blocks

Since both novel haplotype blocks were located within introns, these regions are likely to regulate the expression levels of the genes via transcription factor binding or altering splicing. We investigated each SNP, located within candidate haplotypes, in relation to changes in TFBS affinities and pre-mRNA splicing using the fastSNP web server. While none of the variants were found shown to change ERBB4 or NRG1 splicing directly, alterations TF binding were observed between major and minor alleles. With respect to candidate *ERBB4* region, binding of USF and deltaE, instead of NKX2, was associated with a protective role in schizophrenia, which was due in part by the rs7589006 variant within the 4-SNP haplotype. Similarly, with respect to *NRG1* haplotype block, binding of the TATA, instead of CDXA-E2F-HFH2, had a protective effect, whereas S8-CDXA-NKX2 binding to 3′ end of the block increased risk of schizophrenia in the European population.

In addition to common SNPs investigated (as part of the SNP array data) within susceptible haplotype blocks, many candidate SNPs were in LD. However, they were not assayed in CATIE, GAIN and nonGAIN GWAS. For example, although three datasets encompassed only four SNPs in the 6-kb *ERBB4* region, there were a total of 63 variants in this region, some of which might represent a potential variant with pathogenic consequences. For *NRG1*, we observed a clear difference in transcription factor binding on block between protective and causative haplotypes. Our results suggested that the binding of CDXA/E2F/HFH2 and S8/NKX2/CDXA to the *NRG1* block increased the schizophrenia risk in European populations, and their dissociation and association of TATA and CDP CR transcription factors might be protective. Some of these variants were expected to alter TFBS. Therefore, a combinatorial influence of several SNPs in gene regulation might affect schizophrenia development. To our knowledge, this is the first study to perform an *in silico* functional analysis of variants located within introns in schizophrenia subjects from GWA datasets. This method has given promising results that facilitated our understanding of the functional role of intronic variants. However, future studies should focus on the validation of these results by *in vitro* and *in vivo* studies.

## Supporting Information

Table S1 MAF and p-values of SNPs in *ERBB4* and *NRG1* before and after imputation. p values and minor allele frequencies (MAF) of each polymorphism in ERBB4 and NRG1 haplotype blocks before and after imputation were shown for all three datasets.
(XLSX)

Table S2 Results of haplotype analysis of 6-kb *ERBB4* region after imputation. The haplotype frequencies and p values were calculated using Haploview software.
(XLSX)

Table S3 Results of haplotype analysis of 25-kb *NRG1* region after imputation. The haplotype frequencies and p values of each significant block were calculated using Haploview software.
(XLSX)

Table S4 Rare SNPs in 6-kb Erbb4 and 25-kb NRG1 region that change transcription binding sites. Risk column shows predicted functional effect of each SNP on the gene according to FastSNP analysis decision tree. Wild type and Polymorphic columns show the transcription factors that bind DNA in presence of major and minor allele of each SNP, respectively.
(DOC)
