# Supplementary material for: Discovery, Validation and Characterization of Erbb4 and Nrg1 Haplotypes Using Data from Three Genome-Wide Association Studies of Schizophrenia
Source: PLoS One. 2013 Jan 3;8(1):e53042. doi: 10.1371/journal.pone.0053042 (PMC3536812; doi:10.1371/journal.pone.0053042)
Supplement: Table S4 — Rare SNPs in 6-kb Erbb4 and 25-kb NRG1 region that change transcription binding sites. Risk column shows predicted functional effect of each SNP on the gene according to FastSNP analysis decision tree. Wild type and Polymorphic columns show the transcription factors that bind DNA in presence of major and minor allele of each SNP, respectively. (DOC) [file pone.0053042.s004.doc]

|  |  |  |  | **FastSNP results** | | |
| --- | --- | --- | --- | --- | --- | --- |
| **Chr. Position** | **SNP** | **Location** | **dbSNP allele** | **Risk** | **Wild type** | **Polymorphic** |
| *ERBB4* |  |  |  |  |  |  |
| 212449878 | rs58786592 | intron | A/C | 1-2 | c-ets | Gata1, Gata2 |
| 212451392 | rs12989265 | intron | C/T | 1-2 | - | SRY, Sox5, |
| 212451410 | rs12989282 | intron | C/T | 1-2 | - | SRY |
| 212451460 | rs13012759 | intron | A/G | 1-2 | CdxA | - |
| *NRG1* |  |  |  |  |  |  |
| 32172304 | rs12680997 | intron | C/T | 1-2 | - | SRY |
| 32172391 | rs13278702 | intron | G/T | 1-2 | - | GR |
| 32173468 | rs33932599 | intron | (>6bp) | 1-2 |  |  |
| 32173481 | rs33923195 | intron | (>6bp) | 1-2 | - | Gata1, Gata2 |
| 32174315 | rs66776820 | intron | (>6bp) | 1-2 | TATA | E2F, CdxA, HFH2, Evi1 |
| 32174331 | rs72444140 | intron | (>6bp) | 1-2 |  |  |
| 32174342 | rs3055550 | intron | (>6bp) | 1-2 |  | Evi1 |
| 32174587 | rs10614298 | intron | (>6bp) | 1-2 |  | IRF1 |
| 32176365 | rs35790889 | intron | -/A | 1-2 | Ik2 | - |
| 32177383 | rs71541814 | intron | CA/TG | 1-2 | Oct1 | - |
| 32179035 | rs10684641 | intron | -/CT | 1-2 | HFH-2 | SRY, Evi1 |
| 32179880 | rs72098440 | intron | (>6bp) | 1-2 |  |  |
| 32179893 | rs35859684 | intron | (>6bp) | 1-2 |  |  |
| 32179894 | rs71832406 | intron | (>6bp) | 1-2 | S8 | CdxA |
| 32180378 | rs5890637 | intron | (>6bp) | 1-2 |  |  |
| 32180390 | rs60071647 | intron | (>6bp) | 1-2 |  |  |
| 32180447 | rs6990964 | intron | C/T | 1-2 | - | Gata1 |
| 32180943 | rs9693341 | intron | G/T | 1-2 | MZF1 |  |
| 32181622 | rs72084148 | intron | -/AA | 1-2 |  | CdxA, HNF-3b |
| 32183447 | rs13262178 | intron | C/T | 1-2 | NRF-2 | - |
| 32184330 | rs35960536 | intron | -/T | 1-2 | C/EBPa, C/EBPb, CdxA |  |
| 32185944 | rs7825625 | intron | A/C | 1-2 | HFH-2, CdxA, HNF-3b | - |
| 32186438 | rs56657838 | intron | (>6bp) | 1-2 |  | deltaE |
| 32187323 | rs35638108 | intron | -/G | 1-2 | CdxA | C/EBP |
| 32187487 | rs35422231 | intron | A/C | 1-2 | C/EBP, CdxA | ARP1 |
| 32187503 | rs71512619 | intron | A/C | 1-2 | deltaE, AML-1a | MZF1 |
| 32187511 | rs35727240 | intron | C/T | 1-2 | - | C/ETS |
| 32188459 | rs10676449 | intron | (>6bp) | 1-2 |  | HNF-3b, HFH1 |
| 32188460 | rs34393015 | intron | (>6bp) | 1-2 | Sox5, HFH2 |  |
| 32188480 | rs58045757 | intron | G/T | 1-2 | HNF-3b |  |
| 32188605 | rs60754823 | intron | C/T | 1-2 | - | AML-1a, XFD-3 |
| 32189650 | rs72406944 | intron | -/A | 1-2 | HFH-1 |  |
| 32193522 | rs34158863 | intron | -/G | 1-2 | c-ETS | - |
| 32193545 | rs34782215 | intron | -/G | 1-2 | c-ETS, Elk1 | - |
| 32194531 | rs7000001 | intron | G/T | 1-2 |  |  |
| 32195198 | rs71208174 | intron | (>6bp) | 1-2 | Gata1 | - |
| 32195236 | rs1685114 | intron | A/G | 1-2 |  | Nkx2 |
| 32195289 | rs34464252 | intron | -/C | 1-2 |  | Gata1, MZF1 |
| 32197403 | rs34985716 | intron | G/T | 1-2 | - | Sox5, SRY |
